# Family harmony, emotion-management skills, problematic social media use and individual social responsibility: testing a serial mediation model

**DOI:** 10.1192/bjo.2026.12058

**Published:** 2026-07-31

**Authors:** Mustafa Ercengiz, Samet Makas, Suat Polat, Nurullah Şahin, Murat Yıldırım

**Affiliations:** Department of Educational Sciences, Division of Guidance and Psychological Counselling, Ağrı İbrahim Çeçen University, Türkiye; Department of Educational Sciences, Division of Guidance and Psychological Counselling, Sakarya University, Türkiye; Department of Turkish and Social Sciences Education, Division of Social Studies Education, Ağrı İbrahim Çeçen University, Türkiye; Department of Turkish and Social Sciences Education, Division of Turkish Education, Ağrı İbrahim Çeçen University, Türkiye; Department of Psychology, Faculty of Science and Letters, Ağrı İbrahim Çeçen University, Türkiye

**Keywords:** Family harmony, emotion-management skills, individual social responsibility, problematic social media use, serial mediation model

## Abstract

**Background:**

Family harmony, emotion-management skills, problematic social media use and individual social responsibility are relevant to young adults’ psychosocial functioning, but evidence on their interrelations remains limited, particularly in Turkish samples.

**Aims:**

This study examined whether emotion-management skills and problematic or dysregulated social media use statistically mediated the association between family harmony and individual social responsibility among Turkish young adults.

**Method:**

A cross-sectional online survey was completed by 854 participants (64.1% female) using the Individual Social Responsibility Scale, Family Harmony Scale, Emotion-Management Skills Scale and the Bergen Social Media Addiction Scale, interpreted here as an indicator of problematic or dysregulated social media use symptoms.

**Results:**

Family harmony showed a significant total association with individual social responsibility (*B* = 0.77, 95% CI 0.64 to 0.89) and remained directly associated after the mediators were included (*B* = 0.60, 95% CI 0.47 to 0.72). The total indirect effect was significant (*B* = 0.17, 95% CI 0.13 to 0.22), including specific indirect associations through emotion-management skills (*B* = 0.11, 95% CI 0.07 to 0.15), problematic social media use (*B* = 0.04, 95% CI 0.02 to 0.06) and their serial pathway (*B* = 0.03, 95% CI 0.01 to 0.04).

**Conclusions:**

Family harmony was associated with individual social responsibility directly and through emotion-related and digital-behavioural correlates. Because the data are cross-sectional, findings should be interpreted as associative rather than causal.

Humans develop within social relationships, and their emotions and behaviours can be shaped by close interpersonal contexts such as the family. Among these systems, the family is one of the most proximal and enduring contexts influencing psychological adjustment.^1^ Family harmony, as an indicator of positive relational functioning within the family system, may therefore be closely linked to how individuals manage their emotions and regulate their everyday behaviours.

Extending this family-process perspective to contemporary digital contexts is also important. Family harmony is increasingly expressed not only through face-to-face support and low conflict, but also through shared digital norms, parental and family modelling of technology use, responsiveness during online and offline interactions and the degree to which digital media either supports or disrupts relational availability. Although the present study did not directly assess digital parenting practices, the model can be interpreted within a broader digital family-process perspective in which family relationships and digital behaviours are intertwined rather than separate domains.^2,3^

Empirical evidence suggests that family harmony is linked to healthier emotional functioning and lower levels of dysregulated digital behaviours and other risky behaviours.^4–6^ In particular, individuals from more harmonious family environments tend to report stronger emotion-management skills.^7–9^ This association is significant because emotion management has also been connected to lower levels of problematic social media use.^10–13^ From this perspective, emotion management may be relevant to how family harmony is associated with problematic social media use.

Accordingly, this study tests a cross-sectional indirect-association model in which emotion management is examined as a variable that may statistically account for the association between family harmony and problematic social media use. Given the cross-sectional nature of the data, any indirect effect should be interpreted as theory-informed and statistical rather than as evidence of causality.

## Family harmony and individual social responsibility

Family harmony refers to the perceived quality of family relationships characterised by supportive interactions, low conflict, shared time, mutual help and a sense of responsibility for family affairs. In this sense, it reflects not merely the absence of discord but also the presence of cooperation, emotional support and constructive engagement among family members.^14,15^ Individual social responsibility, in turn, refers to an individual’s awareness of social issues, sensitivity to the needs of others and willingness to act for the welfare of the broader community.^16,17^

In the present manuscript, individual social responsibility is used to denote an integrated responsibility orientation that includes both self-regulatory/personal responsibility and concern for others in social contexts. Although related to prosocial behaviour, civic responsibility and social connectedness, individual social responsibility is broader than discrete helping behaviours and does not necessarily imply formal civic participation or perceived interpersonal belonging. Rather, it captures a general orientation towards respectful, responsible and socially considerate conduct.

From a developmental, family-process and social-capital perspective, harmonious family environments may provide an important relational context in which individuals learn cooperation, reciprocity, empathy, trust and concern for others. Family harmony may therefore be understood as a source of family-based social capital: supportive and low-conflict family relationships can foster shared norms, mutual obligation and interpersonal trust that may generalise beyond the family system.^18,19^ These relational resources are relevant to individual social responsibility because socially responsible behaviour often requires individuals to move beyond self-interest, consider the needs of others and act in ways that support collective well-being. More broadly, Turkish-context evidence indicates that meanings attached to close relational institutions, such as marriage, are associated with psychosocial variables including loneliness and hopelessness, further supporting the relevance of relational meanings to psychological and social functioning.^20^ Empirical findings are broadly consistent with this view. For example, Cheng et al^21^ found that family harmony was positively associated with adolescents’ social responsibility and that this association was partly explained by interdependent self-construal and social trust. More recently, Cheng et al^4^ reported that family harmony and family conflict were linked to adolescents’ social responsibility, with emotion regulation ability showing a significant indirect association. Taken together, these findings suggest that family harmony may be associated with individual social responsibility not only directly, but also indirectly through relevant psychological and relational processes.

Accordingly, within a cross-sectional mediation framework, family harmony can be treated as a relational correlate of individual social responsibility, while the proposed mediator can be examined as a variable through which this association may be statistically patterned. Such a model does not establish temporal or causal order; rather, it tests whether the association between family harmony and individual social responsibility is statistically consistent with an indirect pathway through the proposed mediator.^4,21^

## Indirect associations with emotion-management skills and problematic social media use

Emotion-management skills, often discussed within the broader literature on emotion regulation, refer to the processes through which individuals influence which emotions they experience, when these emotions arise and how these emotions are experienced and expressed.^22^ These skills do not develop in isolation. Family-based emotion socialisation perspectives suggest that the family constitutes a primary interpersonal context in which individuals learn to identify, modulate and express emotions, because parental responses, emotional climate and recurring interaction patterns shape the development of emotion regulation capacities.^23^ Consistent with this perspective, Cheng et al^4^ reported that family harmony was positively associated with adolescents’ emotion regulation ability, whereas family conflict was negatively associated with it; in turn, emotion regulation ability was positively associated with social responsibility. Accordingly, within a cross-sectional framework, it is reasonable to expect that higher family harmony may be associated with stronger emotion-management skills, and that these skills may be one correlate through which the association between family harmony and individual social responsibility is patterned.

Problematic or dysregulated social media use, assessed in this study with the Bergen Social Media Addiction Scale, refers to a pattern of excessive and poorly controlled social media engagement characterised by symptoms such as preoccupation, persistence, conflict and impairment in daily functioning.^24,25^ In the present manuscript, the term problematic social media use is preferred for interpretation because it is less clinically overextended than addiction terminology and is more consistent with the cross-sectional, non-clinical nature of the data. A growing body of research suggests that regulatory processes are closely linked to such problematic use. In a recent systematic review, San Martín Iñiguez et al^26^ concluded that regulatory processes, especially emotion regulation, show a robust negative association with problematic social media use across age groups and cultural settings. Similarly, Elkin et al^27^ found that difficulties in emotion regulation were positively associated with symptoms of problematic social media use, suggesting that individuals who struggle to manage distress may be more likely to rely on social media as a maladaptive coping strategy. Family context also appears relevant in this regard. For example, Qi et al^28^ found that better family functioning was associated with lower problematic social media use among university students. Related evidence also indicates that problematic social media use is connected to broader interpersonal outcomes. In addition, problematic social media use was linked to adolescents’ social connection, and family life satisfaction played a mediating and moderating role in this relationship.^29^ Taken together, these findings suggest that family harmony may be related to lower levels of problematic social media use both directly and indirectly through stronger emotion-management skills.

Although direct evidence linking problematic social media use specifically to individual social responsibility remains limited, adjacent evidence suggests that dysregulated online engagement may undermine positive social functioning. Xu et al^30^ reported a negative association between social networking site addiction and prosocial behaviour, and Liang et al^31^ found that internet addiction negatively predicted later prosocial behaviour through reduced self-control. Given the conceptual overlap between prosocial behaviour and individual social responsibility, these findings provide a cautious rationale for examining problematic social media use as a second mediator. At the same time, digital media should not be viewed as uniformly detrimental. While dysregulated use may displace family interaction and fragment attention, intentional and relationally embedded digital practices may also support connection, coordination and prosocial engagement. Accordingly, in the present cross-sectional serial mediation model, emotion-management skills and problematic social media use were examined as sequential mediators of the association between family harmony and individual social responsibility. This specification should be interpreted as testing whether the observed associations are statistically consistent with an indirect pathway, rather than as evidence of temporal or causal ordering.

## Present study

The present study examines a theory-informed cross-sectional serial mediation model linking family harmony, emotion-management skills, problematic social media use and individual social responsibility. In the proposed ordering, family harmony is conceptualised as an upstream relational and social-capital context, emotion-management skills as a proximal self-regulatory resource and problematic social media use as a downstream digital-behavioural process associated with individual social responsibility. This ordering was derived from family-process, digital family-process, social-capital and emotion-socialisation perspectives, suggesting that supportive family climates may shape emotion-related capacities and shared relational norms, which in turn may be reflected in more regulated patterns of online engagement. Given the cross-sectional design, the model is interpreted as a theoretically ordered set of associations rather than a temporal or causal sequence.

This study, therefore, extends prior work in two ways. First, instead of treating family functioning, emotion regulation, problematic social media use and responsibility-related outcomes as isolated correlates, it evaluates whether these constructs form a coherent relational-emotional-digital pathway. Second, it shifts the outcome focus beyond maladjustment and risk by examining individual social responsibility, a socially relevant and comparatively underexplored positive outcome in this literature.

More specifically, the study tests whether the association between family harmony and individual social responsibility is statistically consistent with both specific indirect pathways (via emotion-management skills and via problematic social media use) and a sequential indirect pathway running from family harmony to emotion-management skills, then to problematic social media use and finally to individual social responsibility. This approach helps clarify not only whether family harmony matters, but also how its association with a socially oriented outcome may be patterned across intrapersonal and digital-behavioural correlates.

Accordingly, the present study aimed to examine whether emotion-management skills and problematic social media use showed specific and sequential indirect associations in the link between family harmony and individual social responsibility among Turkish young adults.

H1: Family harmony will be positively associated with individual social responsibility.

H2: Emotion-management skills will show a significant indirect association in the link between family harmony and individual social responsibility.

H3: Problematic social media use will show a significant indirect association in the link between family harmony and individual social responsibility.

H4: Emotion-management skills and problematic social media use will show a significant serial indirect association in the link between family harmony and individual social responsibility.

## Method

### Participants and procedure

The final analytic sample consisted of 854 young adults aged between 18 and 30 years. Of the participants, 64.1% were female and 35.9% were male. Participants were recruited through convenience sampling because of time, budget and resource limitations. Eligibility criteria required participants to be between 18 and 30 years of age and to complete the Turkish-language questionnaire voluntarily. The survey link was circulated through online networks accessible to the researchers, and individuals who met the age criterion were invited to participate. Data were collected via an online survey administered through Google Forms (Google LLC, Mountain View, CA, USA; https://workspace.google.com/products/forms/). Because the survey was anonymous and self-administered, participants first viewed a brief study information page and proceeded only after providing electronic informed consent. Participation was voluntary, and no financial or academic incentives were offered. Participants were instructed not to provide any identifying information. The exact number of initially accessed survey records was not available in the analysis file. The retained eligible complete-case data-set contained 872 records after applying the age criterion, removing incomplete responses and excluding response records with invalid response patterns. Invalid response patterns were defined as invariant responses across extended scale sections or clearly inconsistent eligibility/demographic entries. Eighteen cases exceeded the Mahalanobis distance cut-off and were removed, resulting in the final analytic sample of 854 participants. Ethical approval was obtained from the Scientific Research Ethics Committee of Ağrı İbrahim Çeçen University (approval no. E-95531838-050.99-67831). The authors assert that all procedures contributing to this work comply with the ethical standards of the relevant national and institutional committees on human experimentation and with the Helsinki Declaration of 1975, as revised in 2013. To evaluate sample adequacy, we conducted an a priori regression-based power analysis in G*Power version 3.1 for Windows (Heinrich Heine University Düsseldorf, Düsseldorf, Germany; https://www.psychologie.hhu.de/arbeitsgruppen/allgemeine-psychologie-und-arbeitspsychologie/gpower).^32^ Assuming a small effect size (*f^2^* = 0.02), *α* = 0.05, power = 0.80 and 3 predictors in the final outcome equation, the minimum required sample size was 550. The final analytic sample of 854 participants therefore exceeded this requirement. Even under a more conservative four-predictor specification, the required sample size remained below the achieved sample size (*N* = 854).

### Measures

#### Individual Social Responsibility Scale^33^

The original Personal and Social Responsibility Questionnaire was developed by Li et al^33^ to assess personal and social responsibility and consists of 14 items rated on a 6-point Likert scale. In the original form, the scale has a two-factor structure representing social responsibility and personal responsibility. The Turkish adaptation was conducted by Filiz and Demirhan.^34^ In the Turkish adaptation study, 1 reverse-coded item was removed because it showed a low item-total correlation, and the final version consisted of 13 items loading on a single factor representing responsibility behaviours. The adapted scale includes no reverse-coded items, and total scores range from 13 to 78, with higher scores indicating greater responsibility. A sample item is ‘I respect others.’ A total-score approach was preferred in the present study because we aimed to model young adults’ overall individual social responsibility orientation as a broad, integrated outcome rather than to compare personal and social responsibility as separate subdimensions. This decision is consistent with the Turkish adaptation study, which supported unidimensional scoring in this language form and cultural context, and it provides a parsimonious indicator that fits the study’s focus on relational, emotional and digital-behavioural correlates of socially considerate conduct. In the adaptation study, the model showed acceptable fit, *χ^2^* (65) = 188.36, incremental fit index (IFI) = 0.98, comparative fit index (CFI) = 0.98, normed fit index (NFI) = 0.96, Tucker–Lewis index (TLI) = 0.97, standardised root mean square residual (SRMR) = 0.047 and root mean square error of approximation (RMSEA) = 0.095. The internal consistency coefficient reported for the Turkish form was 0.925, whereas Cronbach’s alpha was 0.85 in the present study.

#### Family Harmony Scale^14^

This self-report scale consists of five items designed to assess family harmony. Items are rated on a five-point Likert scale ranging from 1 (strongly disagree) to 5 (strongly agree). The scale includes no reverse-coded items. Total scores are obtained by summing all items, with possible scores ranging from 5 to 25; higher scores indicate greater family harmony. A sample item is ‘My family is harmonious’. In the present study, Cronbach’s alpha was 0.87. The Turkish adaptation of the scale was conducted by Duman Kula et al,^35^ who supported its single-factor structure and reported acceptable model fit indices, *χ^2^* (5, *N* = 483) = 17.739, CFI = 0.99, TLI = 0.99, SRMR = 0.016 and RMSEA = 0.073.

#### Emotion-Management Skills Scale^36^

This self-report scale consists of 28 items designed to assess individuals’ perceived emotion-management skills. The scale includes 8 positively worded and 20 negatively worded items and is scored on a 5-point Likert scale. Negatively worded items are reverse-coded, and total scores range from 28 to 140, with higher scores indicating greater emotion-management skills. A sample item is ‘I believe that I generally cope well with my negative emotions’. In the original scale development study, a six-factor structure was identified: verbal expression of emotions, recognising and accepting emotions, expressing emotions as they are, controlling negative bodily reactions, coping and anger management. The six-factor solution explained 48.04% of the total variance, with factor loadings ranging from 0.41 to 0.78. The original study reported a Cronbach’s alpha of 0.83 for the total scale and a test–retest reliability coefficient of 0.81. In the present study, Cronbach’s alpha for the total scale was 0.80.

#### Bergen Social Media Addiction Scale^24^

The scale was developed to measure the six core symptoms commonly discussed in behavioural-addiction frameworks: mood modification, salience, tolerance, conflict, withdrawal and relapse. It includes six items loading on a single factor, each rated on a five-point Likert-type scale ranging from 1 (very rarely) to 5 (very often). A total score is calculated by summing all items, with higher scores indicating greater symptoms of problematic or dysregulated social media use. A sample item is ‘Have you felt an urge to use social media more and more?’ The Turkish adaptation was conducted by Demirci,^37^ who supported the single-factor structure and reported good psychometric properties. The internal consistency coefficient reported in the adaptation study was 0.83. In the present study, Cronbach’s alpha was 0.79. Reported model-fit indices for the Turkish version were NFI = 0.993, TLI = 0.996, CFI = 0.998 and RMSEA = 0.046.

### Data analysis

Before conducting the main analyses, we rechecked all descriptive statistics and model estimates using the raw Statistical Package for the Social Sciences (SPSS) data-set and examined key statistical assumptions. We inspected skewness and kurtosis values to evaluate the distributional properties of the study variables. We assessed internal consistency using Cronbach’s alpha for each observed composite. To evaluate multicollinearity, we examined intercorrelations, tolerance values, variance inflation factors (VIFs) and condition indices. All diagnostic values fell within acceptable thresholds, indicating no evidence of problematic multicollinearity.^38^ We identified multivariate outliers using Mahalanobis distance across the four study variables. Based on the chi-square critical value of 18.47 (*df* = 4, *p* < 0.001), 18 cases exceeded the cut-off and were excluded. The final analytic sample consisted of 854 participants. Because the study relied on self-report data collected from a single source, we also conducted Harman’s single-factor test as a limited diagnostic check for common method bias. We conducted all analyses in IBM SPSS Statistics for Windows, version 21.0 (IBM Corp., Armonk, New York, USA; https://www.ibm.com/products/spss-statistics). We tested the hypothesized serial mediation model using Hayes’ PROCESS macro version 4.2 for SPSS (Model 6; Andrew F. Hayes, University of Calgary, Calgary, Alberta, Canada; https://www.processmacro.org/), specifying family harmony as the predictor, emotion-management skills and problematic social media use as sequential mediators, and individual social responsibility as the outcome variable. We estimated the primary model without demographic covariates, as we aimed to examine the focal theory-driven associations within a parsimonious framework rather than to estimate adjusted population parameters. Accordingly, the coefficients should be interpreted as conditional associations among the focal constructs. As a robustness check, we re-estimated the serial mediation model with gender entered as a covariate in each endogenous equation (emotion-management skills, problematic social media use and individual social responsibility). We estimated direct and indirect effects using 5000 bootstrap resamples^39^ and summarised explained variance (*R^2^*) for each endogenous variable to aid substantive interpretation. We considered an effect statistically significant when its 95% bootstrap confidence interval did not include zero.

## Results

### Preliminary analyses

Table 1 presents the descriptive statistics and bivariate correlations among the study variables. Family harmony was positively associated with individual social responsibility (*r* = 0.42, *p* < 0.001) and emotion-management skills (*r* = 0.25, *p* < 0.001), and negatively associated with problematic social media use (*r* = −0.26, *p* < 0.001). Individual social responsibility was positively associated with emotion-management skills (*r* = 0.38, *p* < 0.001) and negatively associated with problematic social media use (*r* = −0.32, *p* < 0.001). Emotion-management skills were also negatively associated with problematic social media use (*r* = −0.45, *p* < 0.001). Internal consistency was acceptable across the study measures (*α* = 0.87 for family harmony, *α* = 0.85 for individual social responsibility, *α* = 0.80 for emotion-management skills and *α* = 0.79 for problematic social media use). In the outcome equation, VIF values ranged from 1.10 to 1.29, indicating no multicollinearity concern. In Harman’s single-factor test, the first unrotated component accounted for 16.59% of the variance, suggesting that no single factor dominated the covariance structure; however, this result should be interpreted only as a limited diagnostic and not as definitive evidence that common method variance was absent. A gender-adjusted sensitivity analysis yielded the same overall pattern of significant focal associations: all focal direct paths and all specific and serial bootstrapped indirect associations remained statistically significant, the focal coefficients did not meaningfully change in magnitude or direction and the substantive interpretation of the model was unchanged. In terms of explained variance, family harmony accounted for 6.3% of the variance in emotion-management skills (*R^2^* = 0.063), family harmony and emotion-management skills together accounted for 22.3% of the variance in problematic social media use (*R^2^* = 0.223) and the full serial mediation equation explained 27.0% of the variance in individual social responsibility (*R^2^* = 0.270).

**Table 1:** Descriptive statistics and bivariate correlations among variables (*N* = 854) Table 1 long description.

Variable	1	2	3	4
1. Family harmony	**–**			
2. Individual social responsibility	0.42^***^	**–**		
3. Emotion-management skills	0.25^***^	0.38^***^	**–**	
4. Problematic social media use	−0.26^***^	−0.32^***^	−0.45^***^	**–**
Mean	19.62	65.74	89.94	17.07
s.d.	3.75	6.84	12.43	4.94
Skewness	−0.60	−0.51	0.22	0.03
Kurtosis	−0.09	0.65	−0.20	−0.18
Observed score span	17	38	71	24

*** *p* < 0.001 for all bivariate correlations. Values in the final row indicate the observed score span in the present sample.

### Direct effects

**Fig. 1:**
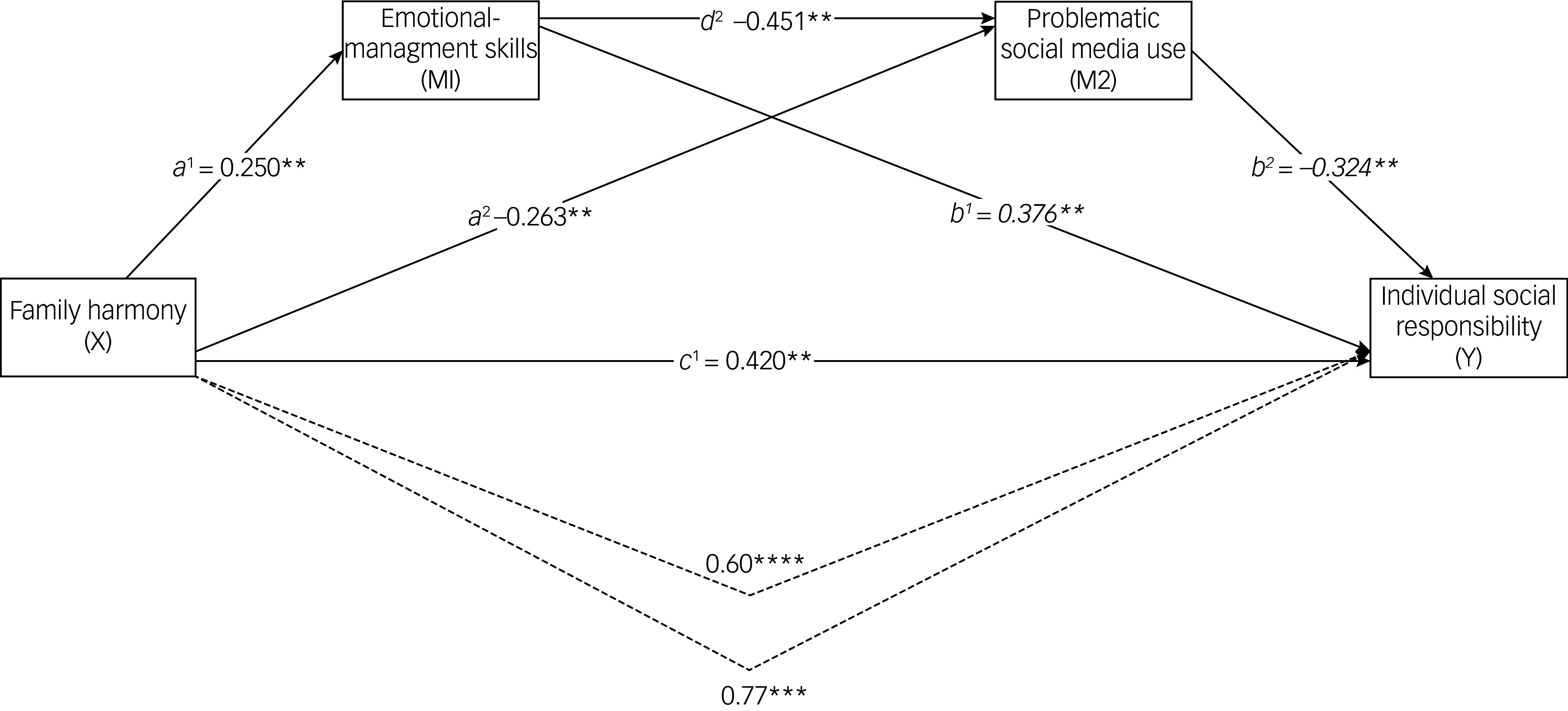
Serial mediation model linking family harmony, emotion-management skills, problematic social media use and individual social responsibility. M1 = emotion-management skills; M2 = problematic social media use; a1 = path from X to M1; a2 = path from X to M2; d21 = path from M1 to M2; b1 = path from M1 to Y; b2 = path from M2 to Y; c′ = direct effect of X on Y; c = total effect of X on Y. Values on the solid paths are standardised coefficients (β); the dashed paths show the unstandardised direct effect (*B* = 0.60) and total effect (*B* = 0.77). All displayed effects are statistically significant at *p* < 0.001.

As shown in Table 2 and Fig. 1, family harmony was positively associated with emotion-management skills (*B* = 0.83, s.e. = 0.11, *t* = 7.57, *p* < 0.001) and individual social responsibility (*B* = 0.60, s.e. = 0.06, *t* = 10.62, *p* < 0.001), while it was negatively associated with problematic social media use (*B* = −.21, s.e. = 0.04, *t* = −5.12, *p* < 0.001). Emotion-management skills were negatively associated with problematic social media use (*B* = −0.16, s.e. = 0.01, *t* = −13.22, *p* < 0.001) and positively associated with individual social responsibility (*B* = 0.13, s.e. = 0.02, *t* = 7.01, *p* < 0.001). In addition, problematic social media use was negatively associated with individual social responsibility (*B* = −0.18, s.e. = 0.05, *t* = −3.99, *p* < 0.001).

**Table 2:** Unstandardised direct effects from the serial mediation model Table 2 long description.

Predictor	Outcome	*B*	s.e.	*t*	*p*
Family harmony	Emotion-management skills	0.83	0.11	7.57	<0.001
Family harmony	Problematic social media use	−0.21	0.04	−5.12	<0.001
Emotion-management skills	Problematic social media use	−0.16	0.01	−13.22	<0.001
Family harmony	Individual social responsibility	0.60	0.06	10.62	<0.001
Emotion-management skills	Individual social responsibility	0.13	0.02	7.01	<0.001
Problematic social media use	Individual social responsibility	−0.18	0.05	−3.99	<0.001

Coefficients are unstandardised and are based on the analytic sample of 854 participants. Test statistics are rounded as displayed.

### Specific and serial indirect effects

We report the results of the serial mediation analyses in Table 3 and depict the overall model in Fig. 1. We found that family harmony had a significant total association with individual social responsibility (*B* = 0.77, 95% CI [0.64, 0.89], *p* < 0.001). After entering emotion-management skills and problematic social media use as sequential mediators, we still observed a significant direct association between family harmony and individual social responsibility (*B* = 0.60, 95% CI [0.47, 0.72], *p* < 0.001). We also found a significant total indirect effect (*B* = 0.17, 95% CI [0.13, 0.22], *p* < 0.001).

**Table 3:** Bootstrap estimates of direct and indirect effects in the serial mediation model Table 3 long description.

Path	Coefficient	95% CI		*p*
		LL	UL	
Family harmony → Emotion-management skills → Individual social responsibility	0.11	0.07	0.15	<0.001
Family harmony → Problematic social media use → Individual social responsibility	0.04	0.02	0.06	<0.001
Family harmony → Emotion-management skills → Problematic social media use → Individual social responsibility	0.03	0.01	0.04	<0.001
Total effect	0.77	0.64	0.89	<0.001
Direct effect	0.60	0.47	0.72	<0.001
Total indirect effect	0.17	0.13	0.22	<0.001

Coefficients are unstandardised. Indirect effects are based on 5000 bootstrap resamples and are reported with percentile 95% confidence intervals. LL, lower limit of the 95% confidence interval; UL, upper limit of the 95% confidence interval.

For the specific indirect effects, we found that family harmony was indirectly associated with individual social responsibility through emotion-management skills (*B* = 0.11, 95% CI [0.07, 0.15], *p* < 0.001). We also observed a significant indirect association through problematic social media use (*B* = 0.04, 95% CI [0.02, 0.06], *p* < 0.001). In addition, we found a significant serial indirect association through emotion-management skills and problematic social media use (*B* = 0.03, 95% CI [0.01, 0.04], *p* < 0.001). Descriptively, the total indirect effect accounted for about 22% of the total association; the pathway through emotion-management skills accounted for the largest share (approximately 14%), followed by the pathway through problematic social media use (approximately 5%) and the serial pathway (approximately 4%). Overall, this pattern of findings suggests that family harmony was associated with individual social responsibility through both direct and indirect pathways, including the sequential pathway involving emotion-management skills and problematic social media use.

## Discussion

The present study examined whether emotion-management skills and problematic social media use were statistically involved in the association between family harmony and individual social responsibility among young adults. Four main findings emerged. First, family harmony was positively associated with individual social responsibility. Second, emotion-management skills showed a significant specific indirect association in this link. Third, problematic social media use also showed a significant specific indirect association. Finally, family harmony was linked to individual social responsibility through a sequential indirect association involving stronger emotion-management skills and lower problematic social media use. Because the direct effect of family harmony remained significant after the indirect paths were modelled, the findings suggest that the association was only partly accounted for by the focal indirect paths. In magnitude terms, the model explained 27% of the variance in individual social responsibility, whereas the upstream equations explained 6 and 22% of the variance in emotion-management skills and problematic social media use, respectively. The total indirect effect also represented about 22% of the total association, indicating that the proposed indirect paths were meaningful but partial rather than exhaustive.

The finding that family harmony was positively associated with individual social responsibility is consistent with prior research showing that supportive family environments are related to stronger social responsibility and prosocial orientations. For example, Cheng et al^21^ reported that family harmony was positively associated with adolescents’ social responsibility, and Cheng et al^4^ similarly found that family harmony was linked to higher social responsibility, whereas family conflict showed the opposite pattern. Together, these findings suggest that harmonious family relationships may provide a relational context in which individuals develop sensitivity to others, reciprocity and concern for the common good. In the present study, this pattern was observed among young adults, extending earlier work by suggesting that the positive association between family harmony and social responsibility may also be relevant beyond early adolescence.

The results also indicated a significant indirect association through emotion-management skills. This finding is theoretically plausible because emotion regulation capacities are shaped within close interpersonal environments, particularly within the family, where individuals learn how to identify, express and modulate emotional experiences.^22,23^ The present result is also consistent with Cheng et al,^4^ who found that emotion regulation ability showed a significant indirect association in the links of family harmony and family conflict with adolescents’ social responsibility. One possible interpretation is that more harmonious family environments may be associated with stronger emotion-management skills, and these skills, in turn, may be linked to more socially responsible orientations. Individuals who are better able to regulate emotional responses may be more capable of considering others’ needs, responding constructively in interpersonal contexts and acting in ways that support collective well-being. At the same time, given the cross-sectional nature of the data, this pathway should be interpreted as a statistically supported indirect association rather than as evidence of temporal or causal ordering.

A second indirect pathway emerged through problematic social media use. Specifically, family harmony was associated with lower problematic social media use, and lower problematic social media use was, in turn, associated with higher individual social responsibility. The first part of this pattern is in line with evidence showing that healthier family functioning is related to lower levels of problematic social media use among young adults.^28^ The second part of the pathway is somewhat less directly established in the literature, but adjacent evidence offers a cautious rationale. A recent systematic review concluded that emotion-regulation-related processes are robustly linked to problematic social media use,^26^ and Elkin et al^27^ similarly reported that difficulties in emotion regulation were positively associated with symptoms of problematic social media use. In addition, Xu et al^30^ found that social networking site addiction was negatively associated with prosocial behaviour, and Liang et al^31^ showed that internet addiction negatively predicted later prosocial behaviour through reduced self-control. Because prosocial behaviour and individual social responsibility overlap conceptually in their emphasis on concern for others and socially beneficial action, these studies provide a cautious basis for interpreting the present indirect effect. Thus, the current findings suggest that problematic social media use may be one correlate through which family harmony is indirectly associated with individual social responsibility.

Most importantly, the serial mediation analysis indicated that family harmony was associated with individual social responsibility through a sequential pathway involving emotion-management skills and problematic social media use. In other words, more harmonious family relationships were associated with stronger emotion-management skills, which were in turn associated with lower problematic social media use, and lower problematic social media use was associated with higher individual social responsibility. This pattern is consistent with the view that family relationships may be linked to social responsibility not only directly, but also through a combination of emotional and behavioural correlates. More specifically, family harmony may be associated with a relational climate that supports emotion management and family-based social capital, and stronger emotion management may coincide with a lower likelihood of relying on social media in maladaptive or dysregulated ways, thereby aligning with higher social responsibility. At the same time, the direct effect of family harmony remained significant after both variables were entered into the model, suggesting that other relevant variables are also likely to contribute. Accordingly, emotion-management skills and problematic social media use should be understood as partial indirect correlates rather than exhaustive accounts of this association.

These findings have several implications. First, they suggest that family harmony may be linked to both lower problematic social media use and higher individual social responsibility in young adulthood. Second, they identify emotion-management skills and problematic social media use as theoretically relevant correlates in this association. Importantly, the digital-use pathway accounted for a smaller portion of the total association than the emotion-management pathway. Thus, problematic social media use should be interpreted as one component of a broader self-regulatory and relational system rather than as the central explanatory mechanism. From an applied perspective, the pattern may help guide family-focused psychoeducational and preventive efforts that incorporate emotion-management skills and healthy digital-use practices. However, digital media should be treated as complex rather than uniformly harmful: dysregulated use may interfere with relational availability, whereas intentional and relationally embedded use may support communication, coordination and social engagement. At the same time, the cross-sectional and self-report nature of the data requires caution. These implications should therefore be treated as hypothesis-generating rather than as evidence that changing family harmony, emotion-management skills or problematic social media use would necessarily alter individual social responsibility. Longitudinal, multi-informant and experimental studies are needed before such change-oriented conclusions can be drawn.

### Theoretical contribution

This study offers three main contributions. First, it conceptualises family harmony as an upstream relational and social-capital context, and evaluates whether its association with individual social responsibility is patterned through a relational-emotional-digital pathway, rather than treating family climate, self-regulation and technology use as unrelated correlates. Second, it extends a literature that has focused predominantly on maladjustment and risk by examining individual social responsibility as a positive, socially relevant outcome. Third, it shows that the association between family harmony and individual social responsibility is statistically consistent with both specific indirect pathways and a sequential pathway involving emotion-management skills and problematic social media use, thereby highlighting a theory-consistent pattern that can inform future longitudinal and intervention-based work.

### Limitations

This study has several limitations. First, the cross-sectional design does not permit temporal ordering or causal inference; therefore, the proposed serial pathway should be understood as theory-informed rather than temporally established. Alternative directional, bidirectional or recursive models are also plausible and should be tested in future longitudinal studies. For example, dysregulated social media use may not only reflect weaker emotion-management capacities but may also contribute over time to poorer emotion regulation, fragmented attention and altered family interaction patterns. Second, all variables were assessed with self-report measures from a single source, which raises the possibility of common-method variance, recall bias and social-desirability effects. Although a Harman-type diagnostic did not suggest domination by a single factor, this check is limited and cannot rule out method bias; therefore, the absence of a dominant single factor should not be interpreted as evidence that common method variance was absent. Because the study did not include a marker variable, multi-informant data or a latent-method-factor approach, common-method bias remains possible. Third, the convenience-sampling strategy and the Turkish young-adult sample limit generalisability to other age groups, cultural contexts and probability-based populations. Fourth, the primary model was estimated in parsimonious form, and although a gender-adjusted sensitivity analysis reproduced the same substantive pattern of results, future research should examine whether the observed pattern holds after accounting for additional factors such as age, socioeconomic context, family digital norms, parental mediation practices and broader psychosocial risk indicators. Future research would benefit from longitudinal and multi-method designs, broader and more diverse sampling frames and the inclusion of additional relevant variables such as parental practices, peer processes, digital family processes, social capital and broader indicators of psychosocial adjustment.

## Data Availability

The raw data supporting the conclusions of this article will be made available by the authors on request.

## Table 1 Long description

The table presents descriptive statistics and bivariate correlations among four study variables: family harmony, individual social responsibility, emotion-management skills, and problematic social media use. The table has four rows and four columns. Column headers are Variable, 1, 2, 3, and 4. Row labels are Family harmony, Individual social responsibility, Emotion-management skills, and Problematic social media use. Each cell contains a value representing the correlation coefficient or descriptive statistic. Family harmony is positively associated with individual social responsibility (0.42) and emotion-management skills (0.25), and negatively associated with problematic social media use (-0.26). Individual social responsibility is positively associated with emotion-management skills (0.38) and negatively associated with problematic social media use (-0.32). Emotion-management skills are negatively associated with problematic social media use (-0.45). The mean values for the variables are 19.62, 65.74, 89.94, and 17.07, respectively. The standard deviations are 3.75, 6.84, 12.43, and 4.94, respectively. Skewness values are -0.60, -0.51, 0.22, and 0.03, respectively. Kurtosis values are -0.09, 0.65, -0.20, and -0.18, respectively. The observed score spans are 17, 38, 71, and 24, respectively.

Navigate back to Table 1.

## Table 2 Long description

A table with six rows and five columns. The columns are labeled Predictor, Outcome, B, s.e., t, and p. The rows present data on the relationships between family harmony, emotion-management skills, problematic social media use, and individual social responsibility. Row 1: Family harmony, Emotion-management skills, 0.83, 0.11, 7.57, <0.001. Row 2: Family harmony, Problematic social media use, -0.21, 0.04, -5.12, <0.001. Row 3: Emotion-management skills, Problematic social media use, -0.16, 0.01, -13.22, <0.001. Row 4: Family harmony, Individual social responsibility, 0.60, 0.06, 10.62, <0.001. Row 5: Emotion-management skills, Individual social responsibility, 0.13, 0.02, 7.01, <0.001. Row 6: Problematic social media use, Individual social responsibility, -0.18, 0.05, -3.99, <0.001.

Navigate back to Table 2.

## Table 3 Long description

A table titled Bootstrap estimates of direct and indirect effects in the serial mediation model. The table has five rows and five columns. The columns are labeled Path, Coefficient, LL, UL, and p. The rows are as follows: Row 1: Family harmony -> Emotion-management skills -> Individual social responsibility, 0.11, 0.07, 0.15, <0.001. Row 2: Family harmony -> Problematic social media use -> Individual social responsibility, 0.04, 0.02, 0.06, <0.001. Row 3: Family harmony -> Emotion-management skills -> Problematic social media use -> Individual social responsibility, 0.03, 0.01, 0.04, <0.001. Row 4: Total effect, 0.77, 0.64, 0.89, <0.001. Row 5: Direct effect, 0.60, 0.47, 0.72, <0.001. Row 6: Total indirect effect, 0.17, 0.13, 0.22, <0.001.

Navigate back to Table 3.
